# Hurricanes, Neighborhood Disadvantage, and Cardiopulmonary Health in US Veterans

**DOI:** 10.1001/jamanetworkopen.2026.7830

**Published:** 2026-04-17

**Authors:** Caryn S. Yip, Peter J. Kaboli, Michael P. Jones, Margaret Carrel, Peter S. Thorne

**Affiliations:** 1 Department of Occupational and Environmental Health, University of Iowa, Iowa City; 2 Center for Comprehensive Access and Delivery Research and Evaluation (CADRE), Iowa City VA Healthcare System, Iowa City; 3 Department of Internal Medicine, University of Iowa Carver College of Medicine, Iowa City; 4 Department of Biostatistics, University of Iowa, Iowa City; 5 School of Earth, Environment, and Sustainability, University of Iowa, Iowa City

## Abstract

**Importance:**

Global climate change is increasing the frequency and severity of hurricanes, which are associated with adverse health outcomes. Veterans may be more susceptible to the health impacts of hurricanes because of prior military exposures.

**Objective:**

To determine whether hurricane exposure is associated with cardiovascular and respiratory events among US veterans.

**Design, Setting, and Participants:**

This retrospective cohort study included US veterans enrolled in Veteran Health Administration (VHA) primary care who lived in areas affected by Hurricane Sandy (2011-2013) or Hurricane Harvey (2016-2018). Data were analyzed between June 2024 and February 2026. Study areas were defined by VHA Veterans Integrated Service Networks (VISNs): VISN 1, 2, 3, 4, and 6 for Hurricane Sandy, and VISNs 16 and 17 for Hurricane Harvey.

**Exposure:**

Living in an area affected by Hurricane Sandy or Hurricane Harvey.

**Main Outcomes and Measures:**

Outcomes of interest were cardiovascular events (CVEs) and respiratory events (REs) that required urgent care and/or emergency department visits or in-patient admission. Associations between hurricane exposure and outcomes were estimated using Andersen-Gill Cox regression models.

**Results:**

Among veterans enrolled in VHA primary care, 1 468 774 lived in the study area during the quarter that Hurricane Sandy occurred, and 1 009 352 lived in the study area during the quarter that Hurricane Harvey occurred. After excluding veterans who were deceased at time of the hurricane or had incomplete data, the analytic cohort included 960 178 veterans exposed to Hurricane Sandy (mean [SD] age, 63 [16] years; 895 646 male [93.30%]) and 654 178 veterans exposed to Hurricane Harvey (mean [SD] age, 60 [16] years; 589 003 male [90.04%]). The analyses included 95 795 CVEs and 120 197 REs for the Hurricane Sandy cohort, and 91 774 CVEs and 98 660 REs for the Hurricane Harvey cohort. Higher levels of neighborhood disadvantage (for healthier veterans with highest levels of disadvantage, hazard ratios [HRs] for CVE were 1.75 [95% CI, 1.63-1.88] for Hurricane Sandy and 1.63 [95% CI, 1.48-1.80] for Hurricane Harvey; HRs for RE were 1.50 [95% CI, 1.43-1.58] for Hurricane Sandy and 1.67 [95% CI, 1.55-1.79] for Hurricane Harvey), age (among healthier veterans aged 60-70 years, HRs for CVE were 2.23 [95% CI, 2.11-2.35] for Hurricane Sandy and 4.12 [95% CI, 3.87-4.39] for those aged ≥70 years for Hurricane Harvey; HRs for RE among sicker veterans were 1.12 [95% CI, 1.08-1.16] for Hurricane Sandy and 1.31 [95% CI, 1.24-1.39] for Hurricane Harvey), and sex (among healthier female veterans, HRs for CVE were 0.52 [95% CI, 0.46-0.59] for Hurricane Sandy and 0.49 [95% CI, 0.44-0.53] for Hurricane Harvey; HRs for RE were 1.38 [95% CI, 1.32-1.43] for hurricane Sandy and 1.36 [95% CI, 1.31-1.41] for Hurricane Harvey) were associated with CVEs and REs in both cohorts. There were no associations between hurricane exposure and CVEs and REs after accounting for baseline differences between exposure regions.

**Conclusions and Relevance:**

This cohort study of 1 614 356 US veterans used an approach that addresses preexisting differences to isolate the association of hurricane exposure with CVEs and REs among veterans. Although changes in HRs specifically attributable to hurricane exposure were not significant, neighborhood disadvantage, advancing age, and sex remained independently associated with acute health events. These findings suggest that disaster preparedness policies should shift from a reactive model toward a longitudinal strategy that addresses baseline social determinants of health and regional environmental hazards.

## Key Points

**Question:**

What are the associations between hurricane exposure and cardiovascular and respiratory events among US veterans?

**Findings:**

In this cohort study of more than 1 million US veterans, exposure to Hurricanes Sandy and Harvey was not significantly associated with cardiopulmonary events. Instead, higher levels of neighborhood disadvantage, age, and sex were factors associated with cardiopulmonary events.

**Meaning:**

This study demonstrates the importance of considering preexisting regional differences and illustrates how prior health status can modify the association between hurricanes and adverse health outcomes.

## Teaser

This cohort study examines whether hurricane exposure is associated with cardiovascular and respiratory events among US veterans.

## Introduction

Global climate change is altering the intensity of hurricanes, causing widespread infrastructure damage and pollution mobilization.^1^ As population density in hurricane-prone areas continues to rise, more communities are at risk of the detrimental effects of hurricanes, including morbidity and mortality, property damage, and displacement.^1,2^ There is growing evidence that hurricane-related stressors are associated with increased risk of adverse cardiovascular events (CVEs) and respiratory events (REs), although studies in general populations report mixed results.^3,4,5,6,7,8,9^ Veterans may be uniquely vulnerable because of military exposures, advancing age,^10^ and higher smoking prevalence.^11,12,13,14^

In October 2012, Hurricane Sandy made landfall in New Jersey and caused extensive damage across the northeastern states,^15^ resulting in 159 deaths.^16^ Hurricane Harvey struck Texas in August 2017 and caused unprecedented flooding and destruction to Houston,^17^ resulting in at least 68 deaths and the displacement of approximately 40 000 people.^16^ Although Hurricanes Sandy and Harvey caused extensive destruction, research on their specific impacts among veterans is limited.^17,18,19,20^ This study utilized patient-level clinical data, residential addresses to more precisely determine exposure, and a novel statistical approach to evaluate the association between these hurricanes and CVEs and REs in US veterans, while accounting for preexisting regional differences.

## Methods

### Study Design and Data Sources

Characteristics of the Veteran Health Administration (VHA) cohort and retrospective longitudinal study design have been reported previously.^21^ Participants were included if they were enrolled in VHA primary care, had a geocoded address on file during the hurricane, and had complete demographic data. Maps from the US Department of Housing and Urban Development and Federal Emergency Management Agency (FEMA) were used to ascertain hurricane exposure.^22,23^ Patient-level clinical and demographic data were obtained from VHA electronic health records. Neighborhood disadvantage data, as measured by the Area Deprivation Index (ADI), were obtained from the University of Wisconsin’s Neighborhood Atlas.^24^ This study was approved by the University of Iowa institutional review board with a waiver of informed consent for retrospective secondary data analysis. This study followed the Strengthening the Reporting of Observational Studies in Epidemiology (STROBE) reporting guidelines.

ESRI’s ArcMap 10.5 was used to link VHA patient data with hurricane data as categorized by 4 groups with increasing exposure: no exposure, public assistance (PA), individual assistance (IA), and flooded with individual assistance (hereafter, flooded) (eFigure 1 in Supplement 1). The flooded category used Housing Damage maps and captured veterans who lived in block groups most affected by the hurricane.^22^ The IA and PA categories captured veterans who lived in counties that either received some IA or only PA from FEMA, respectively.^23^ The IA and PA categories were chosen under the assumption that those living in counties that received IA would be more affected than those living in counties that only received PA.^25^ The no exposure category consisted of veterans in the study area who were not subject to evacuation orders or substantial damage. Tarrant and Dallas counties in Texas, which opened up evacuation shelters, were reclassified as no exposure despite receiving FEMA PA to avoid misclassification of direct storm impact.

The sp package in RStudio software version 4.1.2 (R Project for Statistical Computing) was used to link ADI data to patient-level data.^26,27^ Data regarding prior health status were incorporated in our analysis through the inclusion of the Care Assessments Need (CAN) score, a tool that reflects the likelihood of hospitalization or death within the next year.^28^ CAN score was stratified at or above 80 (sicker) and below 80 (healthy to moderately healthy).

### Outcomes

Our study examined CVEs and REs using *International Classification of Diseases, Ninth Revision* and *International Statistical Classification of Diseases and Related Health Problems, Tenth Revision* codes selected a priori on the basis of medical plausibility (eTable 1 in Supplement 1). To capture the full burden of acute and emergent morbidity associated with hurricane exposure, qualifying events were limited to urgent care or emergency department visits and hospitalizations. This approach ensures the inclusion of high-acuity events requiring immediate care while excluding routine or scheduled follow-up visits.

CVEs included acute myocardial infarction, cardiac dysrhythmias, ischemic heart disease, heart failure, and stroke. REs included chronic obstructive pulmonary disease (COPD), asthma and allied conditions, pneumonia and acute upper respiratory infections, and acute respiratory failure. Fatal events posthurricane were included if the primary reason for the encounter was a qualifying CVE or RE.

### Statistical Analysis

The measures under study were the hazard ratios (HRs) of CVEs and REs among veterans living in the vicinity of hurricanes Sandy and Harvey at the time of their occurrences. Statistical significance was defined as a 95% CI that did not include the null value of 1. The analytical aim was to estimate and test the effects that different hurricane exposure levels have on these event rates compared with a no-exposure level. A veteran’s exposure level was defined as residence in one of the FEMA-defined regions: PA (*Z*_1_ = 1), IA (*Z*_2_ = 1), flooded with IA (*Z*_3_ = 1), and no exposure (*Z*_1_ = *Z*_2_ = *Z*_3_ = 0) used as reference. These regions were determined soon after the hurricane.

We first performed Cox regression based on the Anderson-Gill (AG)^29,30^ marginal model, which allows multiple events per subject. Using only events occurring within 1 year after the hurricane, the covariates were *Z*_1_, *Z*_2_, and *Z*_3_ as binary indicators of future exposure zones (FEZs); ADI quartiles, with the highest quartile indicating highest neighborhood deprivation; age tertiles (Hurricane Sandy, 19-59 years [tertile 1], 60-70 years [tertile 2], and ≥71 years [tertile 3]; Hurricane Harvey, 19-55 years [tertile 1], 56-69 years [tertile 2], and ≥70 years [tertile 3]); and sex; stratified by hurricane and by CAN score (*<*80 [healthy] vs ≥80 [more ill]).

A serious potential problem is that differences may have existed across these regions before the hurricane. To account for these differences, we propose a second set of AG-Cox regression models that covers time periods before and after the hurricane: for veteran *i*, the hazard model is λ_0_(*t*) exp {α′ADI*_i_* + δ′age*_i_* + ηsex*_i_ + *[β_1_ + γ_1_*H*(*t*)]*Z*_1_*_i_* + [β_2_ + γ_2_*H*(*t*)]*Z*_2_*_i_* + [β_3_ + γ_3_*H*(*t*)]*Z*_3_*_i_*}, where time starts 1 year before a hurricane; λ_0_(*t*) is the baseline hazard function; α′ = (α_1_, α_2_, α_3_) are the coefficients for the second, third, and fourth quartiles of ADI; δ′* = *(δ_1_ and δ_2_) are the coefficients for the second and third tertiles of age; and η is the coefficient for female sex. The time-dependent covariate *H*(*t*) = 0 prior to the hurricane and *H*(*t*) = 1 after the hurricane. The γ coefficients allow estimation of the extent to which the hurricane changes preexisting differences among exposure regions. On the basis of the target population of those at risk at the time of the hurricane, and adjusting for ADI, age, and sex, the prehurricane HR of zone *j* vs the no exposure zone is exp(β*_j_*) and the corresponding posthurricane HR is exp(β*_j_* + γ*_j_*), for *j* = 1, 2, 3. Hence, the posthurricane zone *j* vs no exposure zone HR is exp(γ*_j_*) times that of the prehurricane HR. Values greater than 1 indicate a detrimental effect of the hurricane in zone *j*. The null hypothesis of no hurricane effect on zone *j* is a test of *γ_j_* = 0, or equivalently exp(*γ_j_*) = 1. The AG model uses a robust variance estimator to handle correlated event times within subject. Estimates of exp(γ*_j_*) with 95% CIs were then tabulated. This analysis plan addresses preexisting differences in the study population. For completeness, a shared-frailty Cox model was attempted but failed computationally owing to the large sample size.

In addition, HRs for 6 weeks and 6 months after the hurricane were compared with analogous time periods of the previous year to control for the effect modifications of seasonality (eFigures 2-5 in Supplement 1). Further analyses included covariates for self-reported race and ethnicity (eFigures 6 and 7 in Supplement 1). Data on race and ethnicity are included because prior literature has indicated that there are racial and ethnic variations in hurricane exposure and associated health outcomes.^31,32,33^ All statistical analyses were completed in SAS statistical software version 8.3 (SAS Institute) using the PHREG procedure (eAppendix in Supplement 1).^34^

## Results

There were 12 136 407 veterans enrolled in VHA primary care. After excluding veterans who did not have a geocoded address, were deceased at time of the hurricane, lived outside the study area, or had incomplete data, our analytic cohort was 960 178 veterans (Hurricane Sandy) and 654 178 veterans (Hurricane Harvey) (eFigure 8 and eTable 2 in Supplement 1). In the Sandy cohort, the mean (SD) ADI score was 51 (25), the mean (SD) CAN score was 46 (29), and the mean (SD) age at the time of the hurricane was 63 (16) years. In the Harvey cohort, the mean (SD) ADI score was 63 (23), the mean (SD) CAN score was 47 (29), and the mean (SD) age at the time of the hurricane was 60 (16) years. Most participants were male (Sandy, 895 646 men [93.30%]; Harvey, 589 003 men [90.04%]) (Table 1).

**Table 1.** Demographic Characteristics of the Hurricane Sandy and Hurricane Harvey Cohorts

Characteristic	Participants No. (%)	
	Hurricane Sandy (n = 960 178)	Hurricane Harvey (n = 654 178)
Care Assessment Needs score		
<80	777 488 (80.97)	538 272 (82.28)
≥80	182 690 (19.03)	115 906 (18.72)
Mean (SD)	46 (29)	47 (29)
Area Deprivation Index score		
Quartile 1 (least disadvantaged)	168 140 (17.51)	38 111 (5.82)
Quartile 2	318 881 (33.21)	158 907 (24.29)
Quartile 3	282 559 (29.43)	225 024 (34.40)
Quartile 4 (most disadvantaged)	190 598 (19.85)	232 136 (35.49)
Mean (SD)	51 (25)	63 (23)
Age, mean (SD), y	63 (16)	60 (16)
Sex		
Male	895 646 (93.30)	589 003 (90.04)
Female	64 532 (6.70)	65 175 (9.96)
Race and ethnicity^a^		
African American or Black	172 911 (18.95)	150 114 (24.83)
American Indian or Alaska Native, Native Hawaiian or Pacific Islander, Asian	10 148 (1.14)	11 310 (1.87)
Hispanic or Latino	13 062 (1.43)	66 971 (11.08)
Unknown or decline to answer	20 304 (2.22)	28 274 (4.68)
White	690 187 (75.66)	346 909 (57.37)
Hurricane exposure		
No exposure	752 031 (78.32)	477 494 (72.99)
Public assistance	176 996 (18.43)	82 395 (12.59)
Individual assistance	25 931 (2.71)	9861 (1.51)
Flooded with individual assistance	5220 (0.54)	84 428 (12.91)

^a^ Totals for race and ethnicity do not sum to the total number of veterans because of missing data.

In our analysis covering only the posthurricane period, hurricane exposure was significantly associated with CVEs and REs for Hurricane Sandy (Table 2 and Table 3). Among the sickest veterans (CAN ≥80), compared with those living no exposure regions, veterans living in IA (HR, 0.87; 95% CI, 0.76-0.99) and flooded areas (HR, 0.72; 95% CI, 0.56-0.92) had lower HRs for CVEs. Among the healthier veterans (CAN <80), compared with those living no exposure regions, those in IA areas had an HR of 0.78 (95% CI, 0.66-0.91), and those in flooded areas had an HR of 0.89 (95% CI, 0.62-1.28, nonsignificant) for CVE. In contrast, in PA region, among the sickest veterans, the HR for CVE was 1.06 (95% CI, 1.02-1.11), and among the healthier veterans, the HR for CVE was 1.28 (95% CI, 1.20-1.37). Similar results were found in general for REs after Hurricane Sandy.

**Table 2.** Posthurricane Analysis for Cardiovascular Events, Hurricane Sandy and Hurricane Harvey

Variable	HR (95% CI)^a^	
	Hurricane Sandy	Hurricane Harvey
Care Assessment Needs score <80		
Area Deprivation Index score quartile		
1	1 [Reference]	1 [Reference]
2	1.20 (1.11-1.30)	1.16 (1.03-1.32)
3	1.56 (1.44-1.69)	1.47 (1.30-1.65)
4	1.83 (1.69-1.99)	1.72 (1.53-1.94)
Age tertile^b^		
1	1 [Reference]	1 [Reference]
2	2.34 (2.20-2.49)	3.88 (3.58-4.21)
3	1.59 (1.48-1.70)	3.97 (3.66-4.31)
Sex		
Male	1 [Reference]	1 [Reference]
Female	0.49 (0.43-0.57)	0.50 (0.44-0.58)
Hurricane exposure		
No exposure	1 [Reference]	1 [Reference]
Public assistance	1.28 (1.20-1.37)	0.90 (0.83-0.98)
Individual assistance	0.78 (0.66-0.91)	1.07 (0.90-1.26)
Flooded with individual assistance	0.89 (0.62-1.28)	1.35 (1.26-1.44)
Care Assessment Needs score ≥80		
Area Deprivation Index score quartile		
1	1 [Reference]	1 [Reference]
2	1.12 (1.06-1.19)	1.16 (1.04-1.28)
3	1.22 (1.15-1.29)	1.13 (1.02-1.25)
4	1.31 (1.24-1.39)	1.20 (1.09-1.32)
Age tertile^b^		
1	1 [Reference]	1 [Reference]
2	1.93 (1.82-2.04)	2.36 (2.14-2.60)
3	1.87 (1.77-1.98)	2.62 (2.38-3.89)
Sex		
Male	1 [Reference]	1 [Reference]
Female	0.53 (0.48-0.59)	0.49 (044-0.54)
Hurricane exposure		
No exposure	1 [Reference]	1 [Reference]
Public assistance	1.06 (1.02-1.11)	1.04 (0.98-1.11)
Individual assistance	0.87 (0.76-0.99)	0.95 (0.82-1.11)
Flooded with individual assistance	0.72 (0.56-0.92)	1.26 (1.20-1.32)

Abbreviation: HR, hazard ratio.

^a^ This table shows results from Anderson-Gill Cox models that only include data from the posthurricane period.

^b^ Age tertiles for Hurricane Sandy are 19 to 59 years (tertile 1), 60 to 70 years (tertile 2), and 71 years or older (tertile 3). Those for Hurricane Harvey are 19 to 55 years (tertile 1), 56 to 69 years (tertile 2), and 70 years or older (tertile 3).

**Table 3.** Posthurricane Analysis for Respiratory Events, Hurricane Sandy and Hurricane Harvey

Variable	HR (95% CI)^a^	
	Hurricane Sandy	Hurricane Harvey
Care Assessment Needs score <80		
Area Deprivation Index score quartile		
1	1 [Reference]	1 [Reference]
2	1.24 (1.18-1.30)	1.22 (1.11-1.34)
3	1.35 (1.28-1.43)	1.53 (1.39-1.67)
4	1.54 (1.46-1.64)	1.68 (1.53-1.84)
Age tertile^b^		
1	1 [Reference]	1 [Reference]
2	0.82 (0.79-0.85)	1.18 (1.13-1.23)
3	NA	0.85 (0.81-0.90)
Sex		
Male	1 [Reference]	1 [Reference]
Female	1.34 (1.28-1.41)	1.35 (1.28-1.41)
Hurricane exposure		
No exposure	1 [Reference]	1 [Reference]
Public assistance	1.49 (1.43-1.56)	1.19 (1.13-1.25)
Individual assistance	0.69 (0.62-0.78)	0.68 (0.57-0.81)
Flooded with individual assistance	0.52 (0.38-0.72)	0.93 (0.88-0.99)
Care Assessment Needs score ≥80		
Area Deprivation Index score quartile		
1	1 [Reference]	1 [Reference]
2	1.15 (1.09-1.21)	1.08 (0.97-1.20)
3	1.23 (1.17-1.30)	1.16 (1.04-1.28)
4	1.32 (1.25-1.39)	1.20 (1.09-1.33)
Age tertile^b^		
1	1 [Reference]	1 [Reference]
2	1.19 (1.14-1.24)	1.50 (1.41-1.59)
3	0.86 (0.83-0.90)	1.35 (1.27-1.43)
Sex		
Male	1 [Reference]	1 [Reference]
Female	1.12 (1.05-1.19)	1.00 (0.94-1.07)
Hurricane exposure		
No exposure	1 [Reference]	1 [Reference]
Public assistance	1.20 (1.15-1.25)	1.00 (0.95-1.06)
Individual assistance	0.73 (0.65-0.83)	0.75 (0.63-0.90)
Flooded with individual assistance	0.60 (0.45-0.81)	0.94 (0.89-0.99)

Abbreviations: HR, hazard ratio; NA, not applicable.

^a^ This table shows results from Anderson-Gill Cox models that only include data from the post-hurricane period. No data (NA) is shown when there were an insufficient number of events in that strata.

^b^ Age tertiles for Hurricane Sandy are 19 to 59 years (tertile 1), 60 to 70 years (tertile 2), and 71 years or older (tertile 3). Those for Hurricane Harvey are 19 to 55 years (tertile 1), 56 to 69 years (tertile 2), and 70 years or older (tertile 3).

Similarly, the post-Harvey analysis found differences in HRs for CVEs and REs (Table 2 and Table 3). Compared with veterans living in no exposure zones, healthy veterans (HR, 1.35; 95% CI, 1.26-1.44) and sick veterans (HR, 1.26; 95% CI, 1.20-1.32) living in flooded regions had higher HRs for CVEs. In contrast, healthy veterans living in PA areas had a lower HR for CVEs (HR, 0.90; 95% CI, 0.83-0.98). Unlike the Sandy cohort, the HRs between CVEs and REs were inconsistent in the Harvey cohort. Healthy (HR, 0.93; 95% CI, 0.88-0.99) and sick (HR, 0.94; 95% CI, 0.89-0.99) veterans living in flooded areas had lower HRs for REs. Healthy veterans living in PA areas had a higher HR of 1.19 (95% CI, 1.13-1.25).

**Figure 1.**
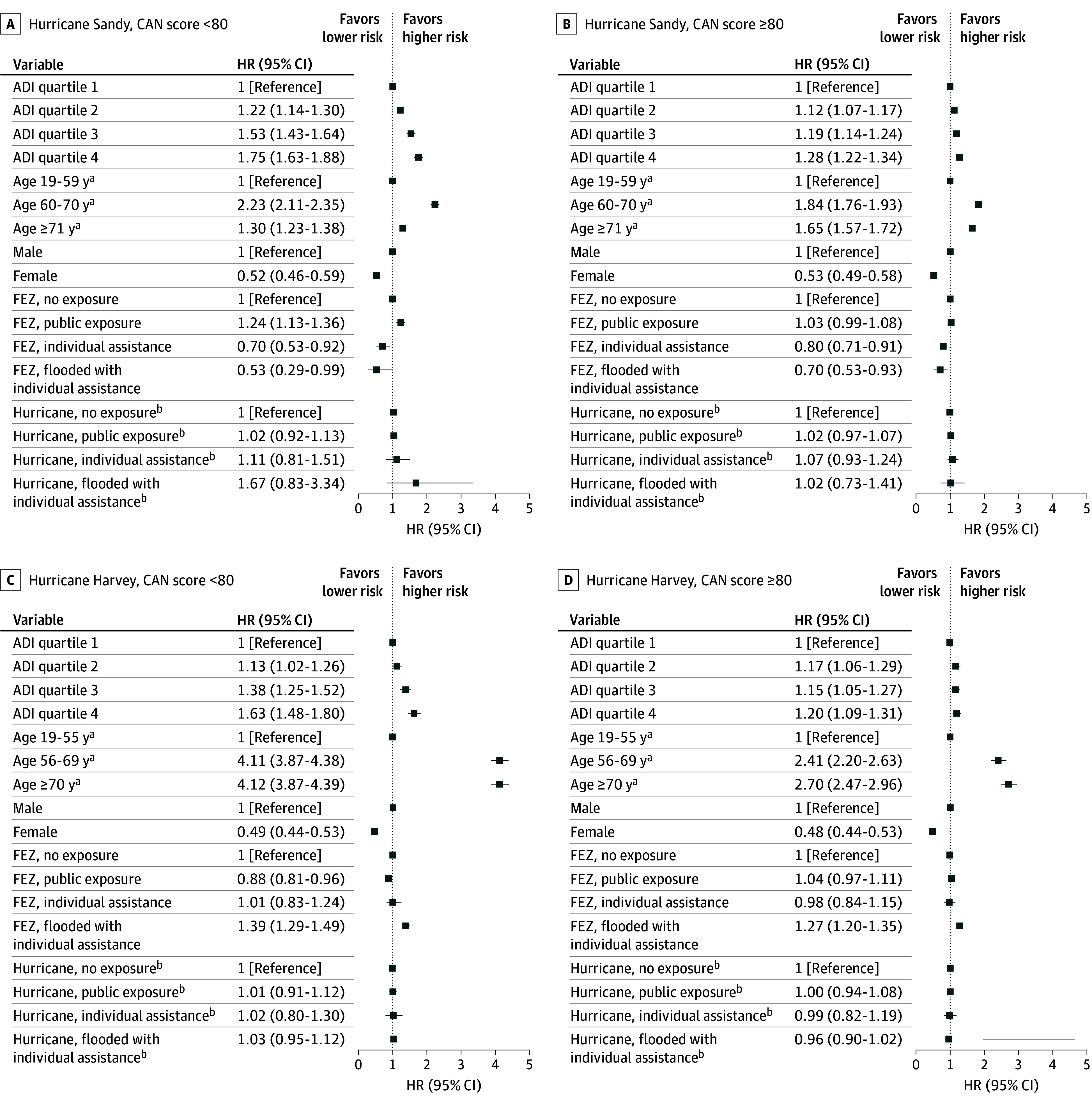
Dot Plots of Risk of Cardiovascular Events Associated With Hurricanes Sandy and Harvey Figure shows hazard ratios (HRs) comparing cardiovascular events at various levels of Area Deprivation Index (ADI) score quartiles, age (tertiles), sex, future exposure zone (FEZ), and hurricane exposure regions, and stratified by Care Assessment Needs (CAN) score (<80 vs *≥*80) for Hurricanes Sandy (A and B) and Harvey (C and D). This model includes data 1 year before and after the hurricane. All analyses are exploratory and no adjustments for multiple comparisons were applied; results should be interpreted as preliminary. ^a^Age ranges for each hurricane differ as each cohort’s age was divided into tertiles. ^b^The lower 4 values are ratios of posthurricane HR and prehurricane HR.

**Figure 2.**
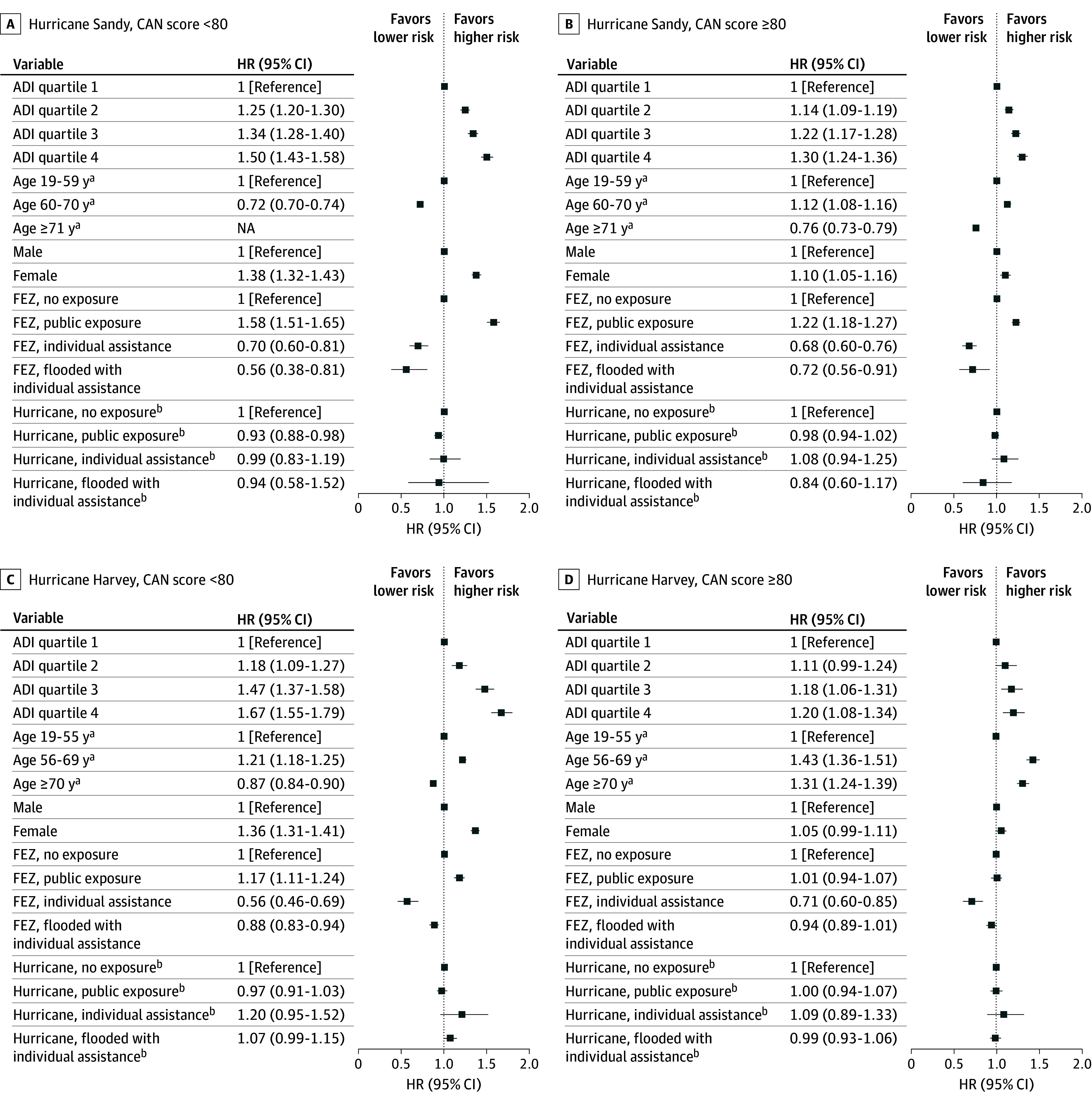
Dot Plots of Risk of Respiratory Events Associated With Hurricanes Sandy and Harvey Figure shows hazard ratios (HRs) comparing respiratory events in various levels of Area Deprivation Index (ADI) score quartiles, age (tertiles), sex, future exposure zone (FEZ), and hurricane exposure regions, and stratified by Care Assessment Needs (CAN) score (<80 vs *≥*80) for Hurricanes Sandy (A and B) and Harvey (C and D). This model includes data 1 year before and after the hurricane. Not applicable (NA) data are shown when there were an insufficient number of events in that strata. All analyses are exploratory and no adjustments for multiple comparisons were applied; results should be interpreted as preliminary. ^a^Age ranges for each hurricane differ as each cohort’s age was divided into tertiles. ^b^The lower 4 values are ratios of posthurricane HR and prehurricane HR.

The 1-year posthurricane analyses show significant HRs for CVEs and REs between the 3 exposure zones and the no exposure zone (Table 2 and Table 3). However, there may have been preexisting differences between the exposure zones that may have biased our analyses and misattributed these results to hurricane exposure. To account for preexisting differences in the hazards of CVEs and REs among the 4 FEZs, our AG-Cox regression models covered a time interval before the hurricane and the corresponding calendar interval after the hurricane. The combined 2-year before-and-after hurricane analyses included 95 795 CVEs and 120 197 REs for Hurricane Sandy, and 91 774 CVEs and 98 660 REs for Hurricane Harvey. As displayed in Figure 1 and Figure 2, living in socioeconomically disadvantaged areas, as measured by ADI quartiles, was associated with higher hazards of CVEs and REs for both hurricanes. This was strikingly true in both order and magnitude for healthier patients (CAN <80; for veterans with ADI quartile 4, HRs for CVE were 1.75 [95% CI, 1.63-1.88] for Hurricane Sandy and 1.63 [95% CI, 1.48-1.80] for Hurricane Harvey; HRs for RE were 1.50 [955 CI, 1.43-1.58] for Hurricane Sandy and 1.67 [95% CI, 1.55-1.79] for Hurricane Harvey), and less so among sicker patients (CAN ≥80) but still significant.

Older age was positively associated with CVEs in both cohorts (Figure 1). For Hurricane Sandy, healthy veterans in the second (HR, 2.23; 95% CI, 2.11-2.35) and third (HR, 1.30; 95% CI, 1.23-1.38) tertiles of age had higher HRs of CVE compared with those aged 59 years and younger. Among sicker veterans, the HRs were 1.84 (95% CI, 1.76-1.93) for those in the second age tertile and 1.65 (95% CI, 1.57-1.72) for those in the third age tertile, compared with younger veterans. For Hurricane Harvey, older age was associated with CVEs (tertile 2 HR, 4.11 [95% CI, 3.87-4.38]; tertile 3 HR, 4.12 [95% CI, 3.87-4.39]). The association was attenuated among the sicker group.

In contrast, age associations for REs were inconsistent. Among healthy Hurricane Sandy veterans, the second tertile had a lower HR (0.72; 95% CI, 0.70-0.74), whereas sicker veterans showed a higher HR for the second tertile (1.12; 95% CI, 1.08-1.16), but a lower HR for the third (0.76; 95% CI, 0.73-0.79). For Hurricane Harvey, healthy veterans in the second tertile had a higher HR (1.21; 95% CI, 1.18-1.25), whereas the HR for the third tertile was lower (HR, 0.87; 95% CI, 0.84-0.90). Among sicker Hurricane Harvey veterans, age was consistently associated with higher REs (56-69 years HR, 1.43 [95% CI, 1.36-1.51]; age ≥70 years HR, 1.31 [95% CI, 1.24-1.39]).

Female veterans had lower HRs for CVEs compared with male veterans for both cohorts (Figure 1). For Hurricane Sandy, healthy (HR, 0.52, 95% CI, 0.46-0.59) and sicker (HR, 0.53; 95% CI, 0.49-0.58) veterans had similar HRs. The Harvey cohort showed a similar association (healthy veterans HR, 0.49 [95% CI, 0.44-0.53]; sicker veterans HR, 0.48 [95% CI, 0.44-0.53]). In contrast, female veterans had higher HRs for REs for both cohorts (Figure 2). Healthier female veterans in our Sandy cohort had an increased HR of 1.38 (95% CI, 1.32-1.43) for REs. The association was attenuated among sicker veterans (HR, 1.10; 95% CI, 1.05-1.16). Similar results were found in our Harvey cohort (HR, 1.36 [95% CI, 1.31-1.41] among healthier veterans vs 1.05 [95% CI, 0.99-1.11] among sicker veterans).

After ADI, age, and sex, the key covariates were the FEZ indicators that represent preexisting effects of regions defined by FEMA-derived posthurricane intensities and the hurricane-by-FEZ interaction, as defined by FEZ × *H*(*t*). The associations of the hurricanes with CVEs and REs (year 2 vs year 1) were assessed via FEZ-by-hurricane interactions, as ratios of HRs. After accounting for preexisting differences among the FEZ, associations between hurricane exposure and CVEs and REs were null. For example, healthier veterans alive at the time of Hurricane Harvey and residing in areas later flooded had a prehurricane CVE HR of 1.39 (95% CI, 1.29-1.49) compared with regions later unexposed. After the hurricane, these same veterans’ CVE HR was only 1.03 (95% CI, 0.95-1.12, nonsignificant) times greater than before the hurricane (Figure 1).

## Discussion

To our knowledge, this cohort study is the first to examine the associations between Hurricanes Sandy and Harvey and CVEs and REs at this granularity, while accounting for preexisting differences between exposure groups. After accounting for these factors, hurricane exposure itself was not significantly associated with CVE or RE risk among US veterans. Instead, neighborhood disadvantage (ADI), advancing age, and sex emerged as the primary factors associated with posthurricane morbidity.

Although our models were optimized to estimate the independent effect of hurricane exposure, the consistent associations observed for secondary covariates, most notably ADI, merit attention. Although the estimates for ADI, age, and sex should be interpreted as adjustment factors rather than primary effects, their robust associations with CVEs and REs emphasize the persistent role of neighborhood-level stressors, advancing age, and sex-based disparities in disaster-prone areas.

Our results support prior literature^35,36,37^ indicating that neighborhood characteristics and socioeconomic status are inextricably linked to cardiovascular and respiratory health. The positive association between ADI and CVE and RE remained consistent across cohorts and time periods, although the association was attenuated among the sickest veterans. This attenuation suggests that for sicker individuals, the benefits of comprehensive VHA health care access may partially offset the risks posed by neighborhood disadvantage.

Our analysis revealed significant sex-based disparities in posthurricane morbidity, with female veterans exhibiting a markedly lower hazard of CVEs, but an elevated hazard of REs compared with male veterans. Although the lower CVE risk aligns with the traditionally observed delayed onset of cardiovascular disease in women,^38^ this finding should be interpreted with caution. Substantial evidence suggest that the perceived lower risk may be partially associated with systemic underdiagnosis and undertreatment of cardiovascular disease in women.^39^ Conversely, the elevated HRs for REs among female veterans is consistent with research indicating that women experience higher rates of asthma and COPD exacerbations when exposed to environmental triggers.^40^ This vulnerability likely stems from a combination of biological factors, such as immunological and hormonal factors,^40^ and behavioral factors, including gendered posthurricane division of labor that increase women’s domestic exposure to indoor mold and endotoxins.^41^

Although advancing age was a robust variable associated with CVEs and REs, its influence is considerably more nuanced than that of ADI. As expected, older age was consistently and positively associated with CVEs across both the Sandy and Harvey cohorts. This aligns with the well-established pathophysiology of cardiovascular disease, where age-related arterial stiffening and accumulated stressors heighten vulnerability to acute triggers, such as a hurricane.^42^ The association between age and REs was inconsistent, particularly among healthier veterans. The observed protective effect for REs among older veterans suggests a prominent healthy survivor effect, especially among veterans in the highest age tertile. Individuals who reach advanced age while maintaining a low CAN score likely represent a resilient subpopulation.^43^

Geographic variations in HRs are likely to reflect regional baseline health disparities rather than hurricane impact alone. In the Sandy cohort, higher HRs in PA areas (eg, eastern West Virginia) relative to flooded coastal zones (eg, New York City and coastal New Jersey) likely reflect higher underlying comorbidity rates in rural Appalachia.^44,45^

In the Harvey cohort, elevated risk for CVEs in flooded regions aligns with the high prevalence of heart disease and stroke historically reported along the Texas Gulf Coast.^44,46^ Conversely, the lower HRs for REs in flooded urban centers may be associated with the urban advantage, whereby metropolitan counties often exhibit lower COPD hospitalization rates than rural counties.^47^ This is further explained by the elevated baseline RE risk in the no exposure and PA groups, which overlap with the Permian Basin and Eagle Ford Shale formations. In these regions, intensive oil and gas extraction is a known factor associated with air pollution and respiratory morbidity.^48,49^

There is growing evidence that hurricane exposure may lead to an increased risk of CVEs.^4,8,50,51^ This may be due to psychosocial stress and/or a disruption in transportation and health care services.^51^ While many studies report increased risk of hospitalization for CVEs, others have observed no significant changes in rates after hurricanes.^3,50,52^ Our study found that hurricane exposure alone was not significantly associated with CVEs.

Flooding and extreme precipitation can lead to exposure to molds and fungi, which is associated with the development and/or exacerbation of COPD, asthma, and allergic rhinitis.^53,54^ Previous collaborative work^6^ conducted by our group in the aftermath of Hurricane Katrina showed that flood-damaged homes had high levels of mold and endotoxins. Notably, we observed a nonsignificant, but modest increase in RE risk among healthy veterans in Harvey’s most impacted areas. We hypothesize that healthier veterans were able to participate in the recovery of their homes after flooding from Harvey, thus exposing themselves to increased levels of mold and endotoxins. The absence of a similar trend in the Sandy cohort may be due to differences in hurricane behavior; although Hurricane Harvey caused unprecedented flooding in the Houston area, rainfall totals from Sandy were limited as the storm encountered a wedge of dry air right before landfall.^17,18^

### Strengths and Limitations

The greatest strength of this research was the use of high-granularity VHA data that linked individual-level exposure and covariate data with individual-level outcomes. This allowed use of an innovative Cox regression model in which individuals have different rates of health events before and after the storm, reducing baseline bias. This study utilized administrative data, which are free of self-reporting bias. In addition, our large sample size provided sufficient power to evaluate CVEs and REs at various time points.

A substantial limitation of this study is the potential for underascertainment of acute events, as our data only captured care delivered within the VHA system. Veterans may have sought treatment at non-VHA facilities, particularly if local VHA facilities were closed or inaccessible due to storm damage. Because such disruptions were most severe in flooded areas, this missing data likely introduced a bias toward the null, potentially masking a significant association between hurricane exposure and CVEs and REs. However, we believe this risk is partially mitigated by our focus on veterans enrolled in VHA primary care, a group that predominantly utilizes the VHA for acute needs and by including records of visits to all VHA facilities, not just those in our study areas. In addition, there is potential for exposure misclassification for participants experiencing flooding, but who lived in a census block determined by FEMA to have fewer than 10 damaged homes. Since these individuals would be categorized into lower-intensity exposure groups, this likely resulted in a bias toward the null.

## Conclusions

In this cohort study of 1 614 356 US veterans, we utilized a novel approach that addresses preexisting differences to isolate the association of hurricane exposure with CVEs and REs among veterans. Although changes in HRs specifically attributable to hurricane exposure were not significant, neighborhood disadvantage, advancing age, and sex remained robust factors independently associated with acute health events. These findings suggest that disaster preparedness policies should shift from a reactive model toward a longitudinal strategy that addresses baseline social determinants of health and regional environmental hazards. As climate change continues to worsen the impacts of hurricanes in the US, building resiliency in communities most vulnerable to their effects is especially salient.

## Notes

Supplement 1. eTable 1. *ICD* Codes for Health Outcomes of Interest eFigure 1. Map of Study Area, Hurricane Exposure, and ADI eFigure 2. Cardiovascular Events, 6 Weeks eFigure 3. Respiratory Events, 6 Weeks eFigure 4. Cardiovascular Events, 6 Months eFigure 5. Respiratory Events, 6 Months eFigure 6. Cardiovascular Events (Including Race/Ethnicity), 1 Year eFigure 7. Respiratory Events (Including Race/Ethnicity), 1 Year Appendix. Code Stubs and Variable Definitions eFigure 8. STROBE Cohort Exclusion Diagram eTable 2. Full Cohort v. Analytical Cohort Comparison

Supplement 2. Data Sharing Statement
