# Supplementary material for: Hurricanes, Neighborhood Disadvantage, and Cardiopulmonary Health in US Veterans
Source: JAMA Netw Open. 2026 Apr 17;9(4):e267830. doi: 10.1001/jamanetworkopen.2026.7830 (PMC13090845; doi:10.1001/jamanetworkopen.2026.7830)
Supplement: Supplement 1. — eTable 1. ICD Codes for Health Outcomes of Interest eFigure 1. Map of Study Area, Hurricane Exposure, and ADI eFigure 2. Cardiovascular Events, 6 Weeks eFigure 3. Respiratory Events, 6 Weeks eFigure 4. Cardiovascular Events, 6 Months eFigure 5. Respiratory Events, 6 Months eFigure 6. Cardiovascular Events (Including Race/Ethnicity), 1 Year eFigure 7. Respiratory Events (Including Race/Ethnicity), 1 Year Appendix. Code Stubs and Variable Definitions eFigure 8. STROBE Cohort Exclusion Diagram eTable 2. Full Cohort v. Analytical Cohort Comparison [file jamanetwopen-e267830-s001.pdf]

# Supplemental Online Content

Yip CS, Kaboli PJ, Jones MP, Carrel M, Thorne PS. Hurricanes, neighborhood disadvantage, and cardiopulmonary health in US veterans. *JAMA Netw Open*. 2026;9(4):e267830. doi:10.1001/jamanetworkopen.2026.7830

**eTable 1.** *ICD* Codes for Health Outcomes of Interest
**eFigure 1.** Map of Study Area, Hurricane Exposure, and ADI
**eFigure 2.** Cardiovascular Events, 6 Weeks
**eFigure 3.** Respiratory Events, 6 Weeks
**eFigure 4.** Cardiovascular Events, 6 Months
**eFigure 5.** Respiratory Events, 6 Months
**eFigure 6.** Cardiovascular Events (Including Race/Ethnicity), 1 Year
**eFigure 7.** Respiratory Events (Including Race/Ethnicity), 1 Year
**Appendix.** Code Stubs and Variable Definitions
**eFigure 8.** STROBE Cohort Exclusion Diagram
**eTable 2.** Full Cohort v. Analytical Cohort Comparison

This supplemental material has been provided by the authors to give readers additional information about their work.

© 2026 Yip CS et al. *JAMA Network Open*.

eTable 1. *ICD* codes for Health Outcomes of Interest

| **Health Outcome** | ***ICD-9* Code** | ***ICD-10* Code** |
|---|---|---|
| Cardiovascular Events (CVE) | | |
| Acute Myocardial Infarction | 410 | I21 |
| Cardiac Dysrhythmia | 427 | I46-I49 |
| Ischemic Heart Disease | 410-414 | I20-I25 |
| Heart Failure | 428 | I50 |
| Stroke | 430-438 | I60-I69 |
| Respiratory Events (REs) | | |
| COPD, including Asthma and Allied Conditions | 466, 490-493, 496 | J20-21, J40-45 |
| Pneumonia and Acute Upper Respiratory Infection | 460-466, 480-486 | J00-J06, J12-J18 |
| Acute Respiratory Failure | 518.81, 518.82 | J96, R06.03 |

© 2026 Yip CS et al. *JAMA Network Open*.

eFigure 1. Map of Study Area, Hurricane Exposure, and ADI

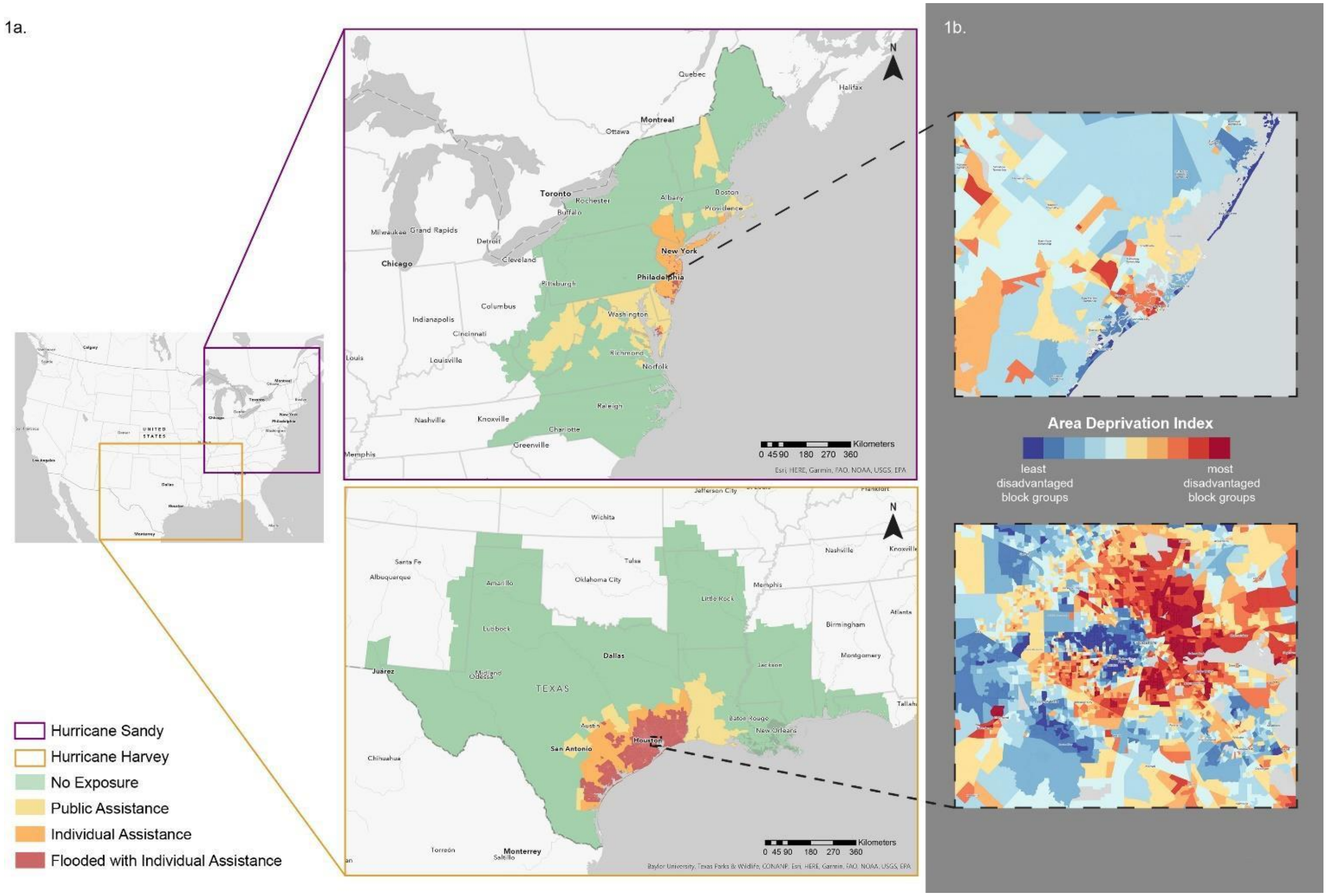


Caption:
1a. Maps of the study area and exposure categories. The purple-bound box shows the study area for Hurricane Sandy while the gold-bound box shows the study area for Hurricane Harvey. The exposure categories shown in these maps are used for both the FEZ (Future Exposure Zone) and Hurricane variables.
1b. A close-up map showing deciles of Area Deprivation Index (ADI) of an area that was flooded after Hurricane Sandy and Hurricane Harvey. These maps show the large variability in deprivation between neighborhoods. For our analysis, we re-coded ADI into quartiles (cutoffs at 25, 50, and 75).

© 2026 Yip CS et al. *JAMA Network Open*.

eFigure 2. Cardiovascular Events, 6 Weeks

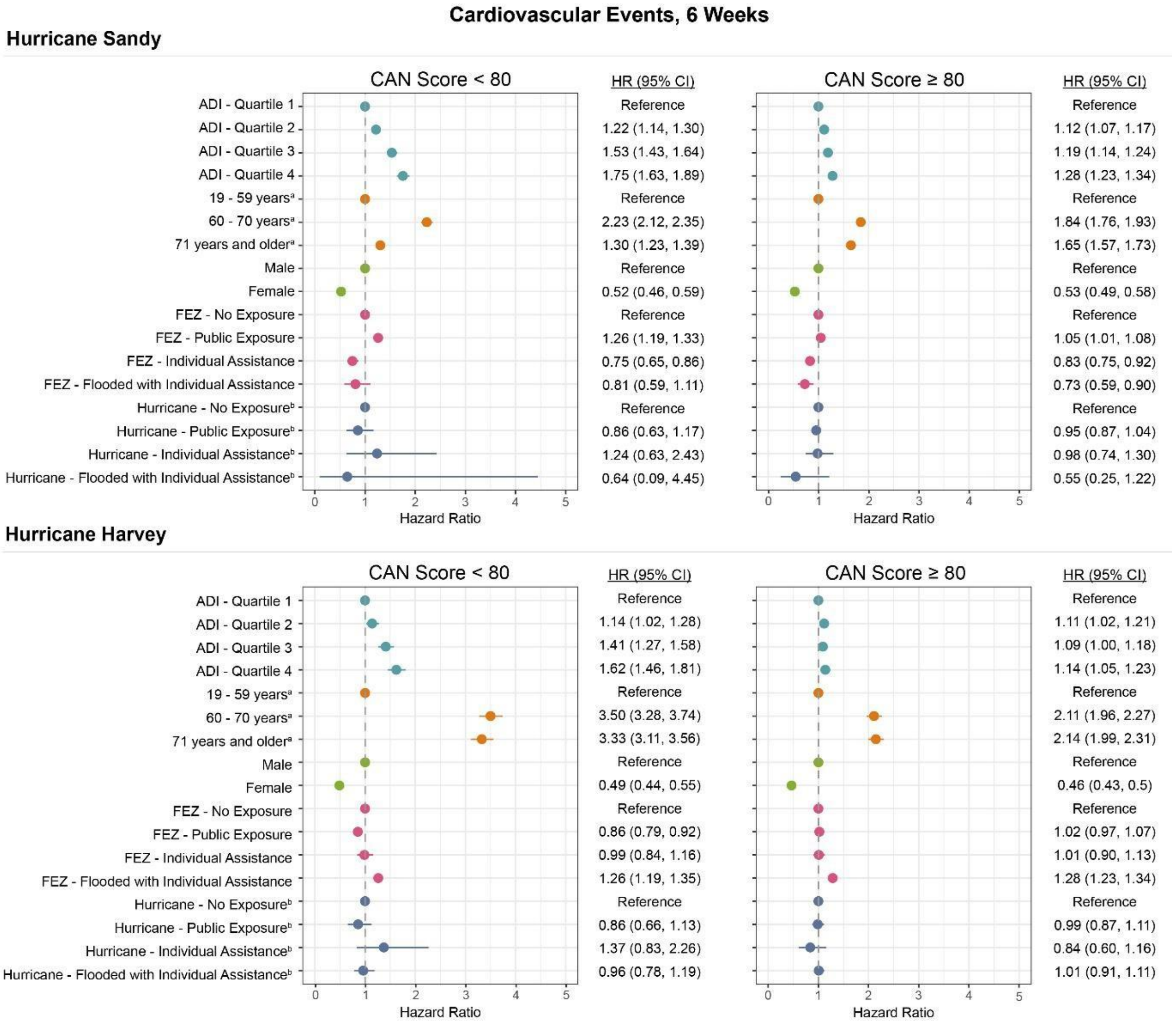


eFigure 2. Hazard ratios (95% confidence intervals) comparing CVEs in various levels of ADI (quartiles), age (tertiles), sex, FEZ (Future Exposure Zone) and hurricane exposure regions, and stratified by CAN score (< 80, ≥ 80) for 6-weeks post hurricane compared to the analogous time period from the previous year.

[a] Age ranges for each hurricane differ as each cohort's age was divided into tertiles.

[b] The lower four values are ratios of post-hurricane HR and pre-hurricane HR.

© 2026 Yip CS et al. *JAMA Network Open*.

eFigure 3. Respiratory Events, 6 Weeks

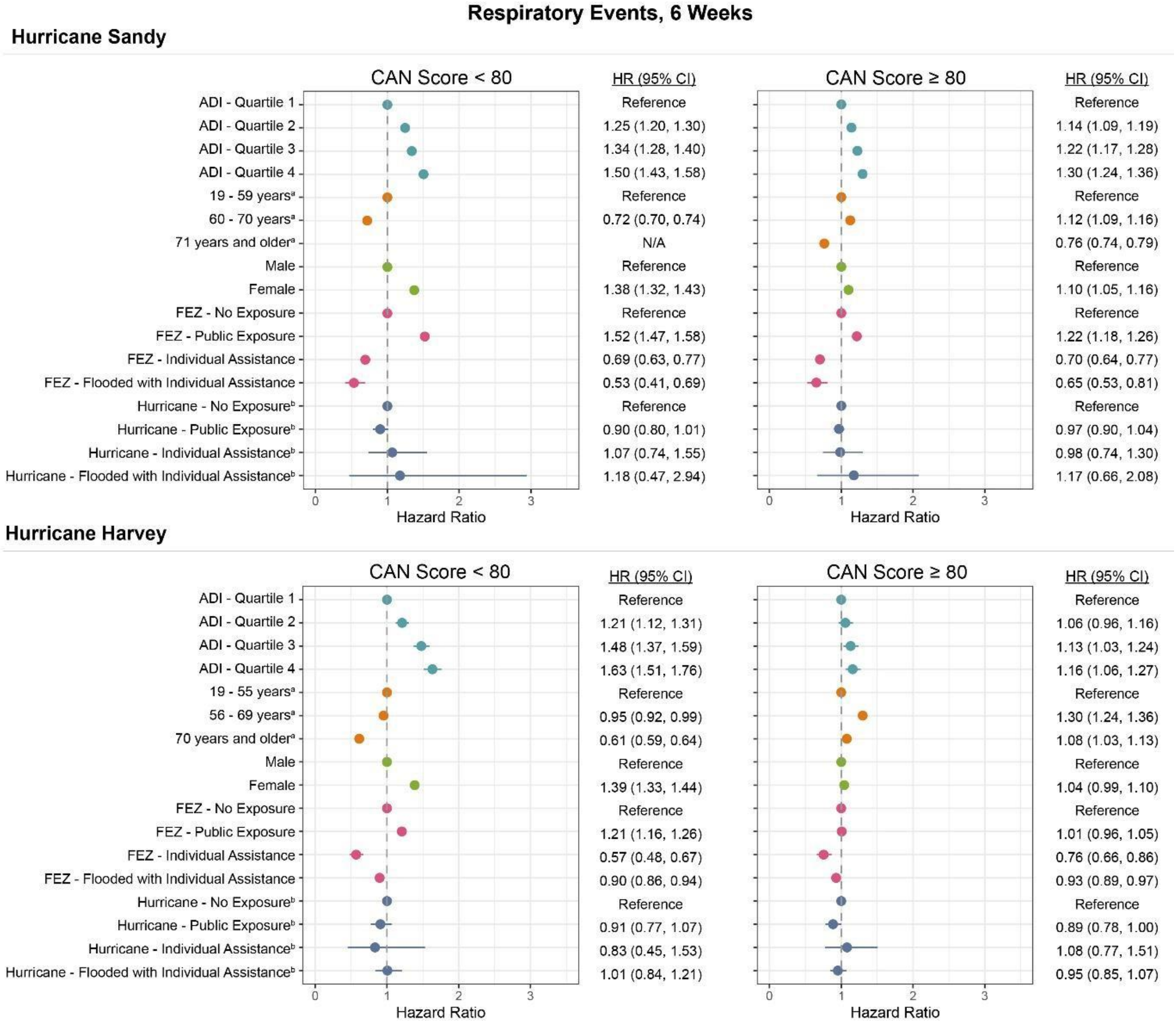


eFigure 3. Hazard ratios (95% confidence intervals) comparing REs in various levels of ADI (quartiles), age (tertiles), sex, FEZ (Future Exposure Zone) and hurricane exposure regions, and stratified by CAN score (< 80, ≥ 80) for 6-weeks post hurricane compared to the analogous time period from the previous year. No applicable data (N/A) is shown when there were insufficient events in that strata.

[a] Age ranges for each hurricane differ as each cohort's age was divided into tertiles.

[b] The lower four values are ratios of post-hurricane HR and pre-hurricane HR.

© 2026 Yip CS et al. *JAMA Network Open*.

eFigure 4. Cardiovascular Events, 6 Months

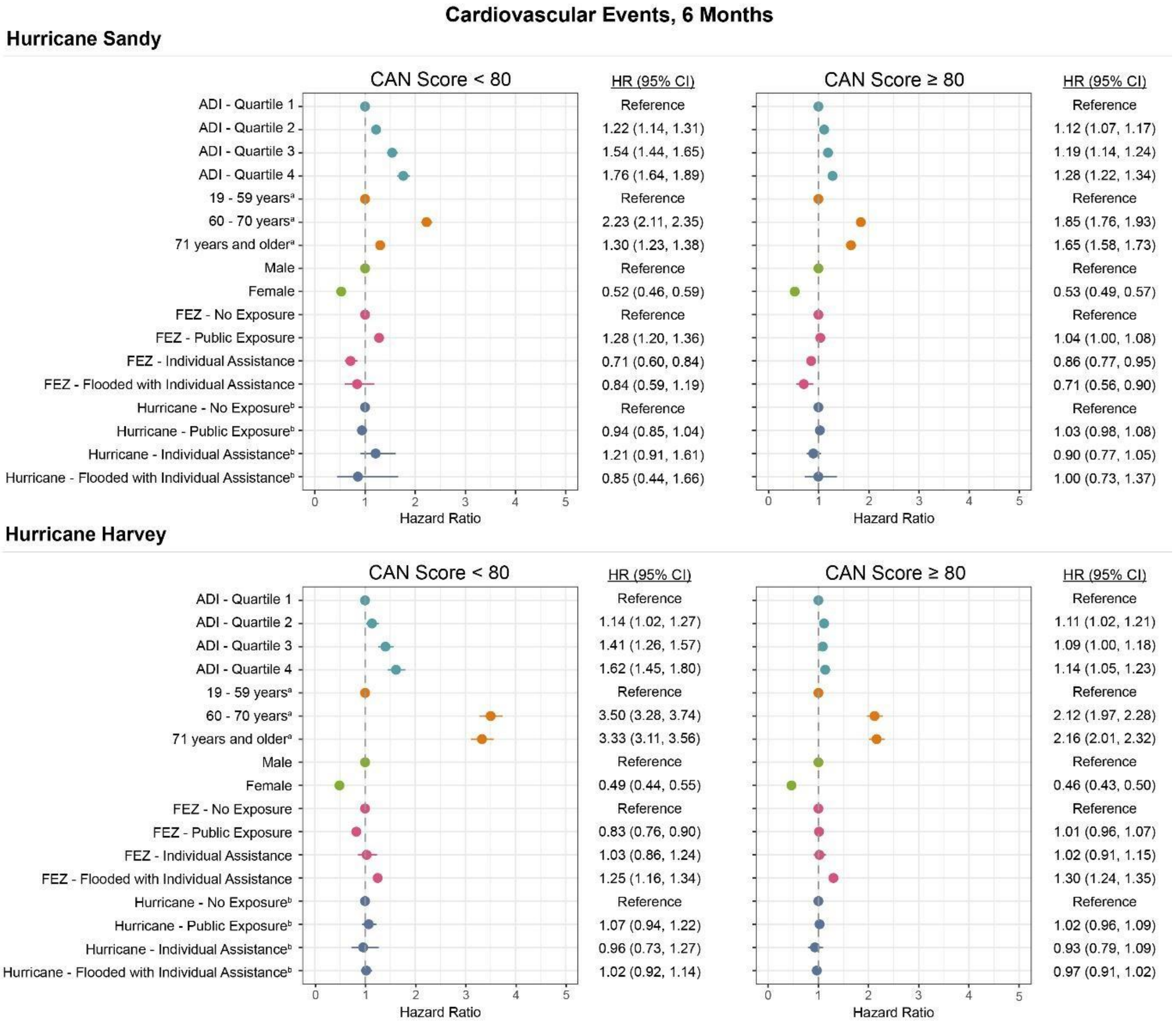


eFigure 4. Hazard ratios (95% confidence intervals) comparing CVEs in various levels of ADI (quartiles), age (tertiles), sex, FEZ (Future Exposure Zone) and hurricane exposure regions, and stratified by CAN score (< 80, ≥ 80) for 6-months post hurricane compared to the analogous time period from the previous year.

[a] Age ranges for each hurricane differ as each cohort's age was divided into tertiles.

[b] The lower four values are ratios of post-hurricane HR and pre-hurricane HR.

© 2026 Yip CS et al. *JAMA Network Open*.

eFigure 5. Respiratory Events, 6 Months

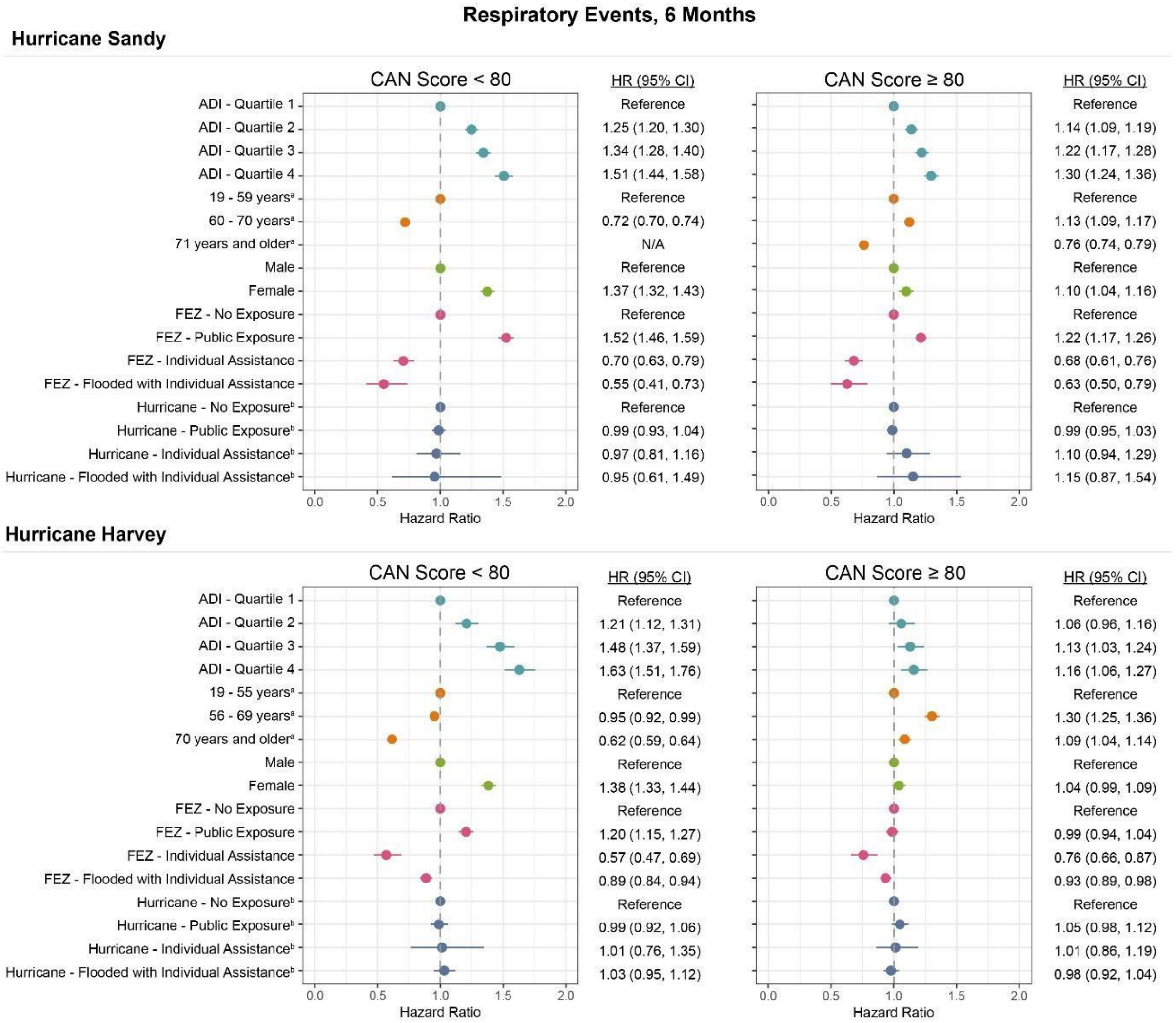


eFigure 5. Hazard ratios (95% confidence intervals) comparing REs in various levels of ADI (quartiles), age (tertiles), sex, FEZ (Future Exposure Zone) and hurricane exposure regions, and stratified by CAN score (< 80, ≥ 80) for 6-months post hurricane compared to the analogous time period from the previous year. No applicable data (N/A) is shown when there were insufficient events in that strata.

[a] Age ranges for each hurricane differ as each cohort's age was divided into tertiles.

[b] The lower four values are ratios of post-hurricane HR and pre-hurricane HR.

© 2026 Yip CS et al. *JAMA Network Open*.

eFigure 6. Cardiovascular Events, Including Race/Ethnicity, 1 Year

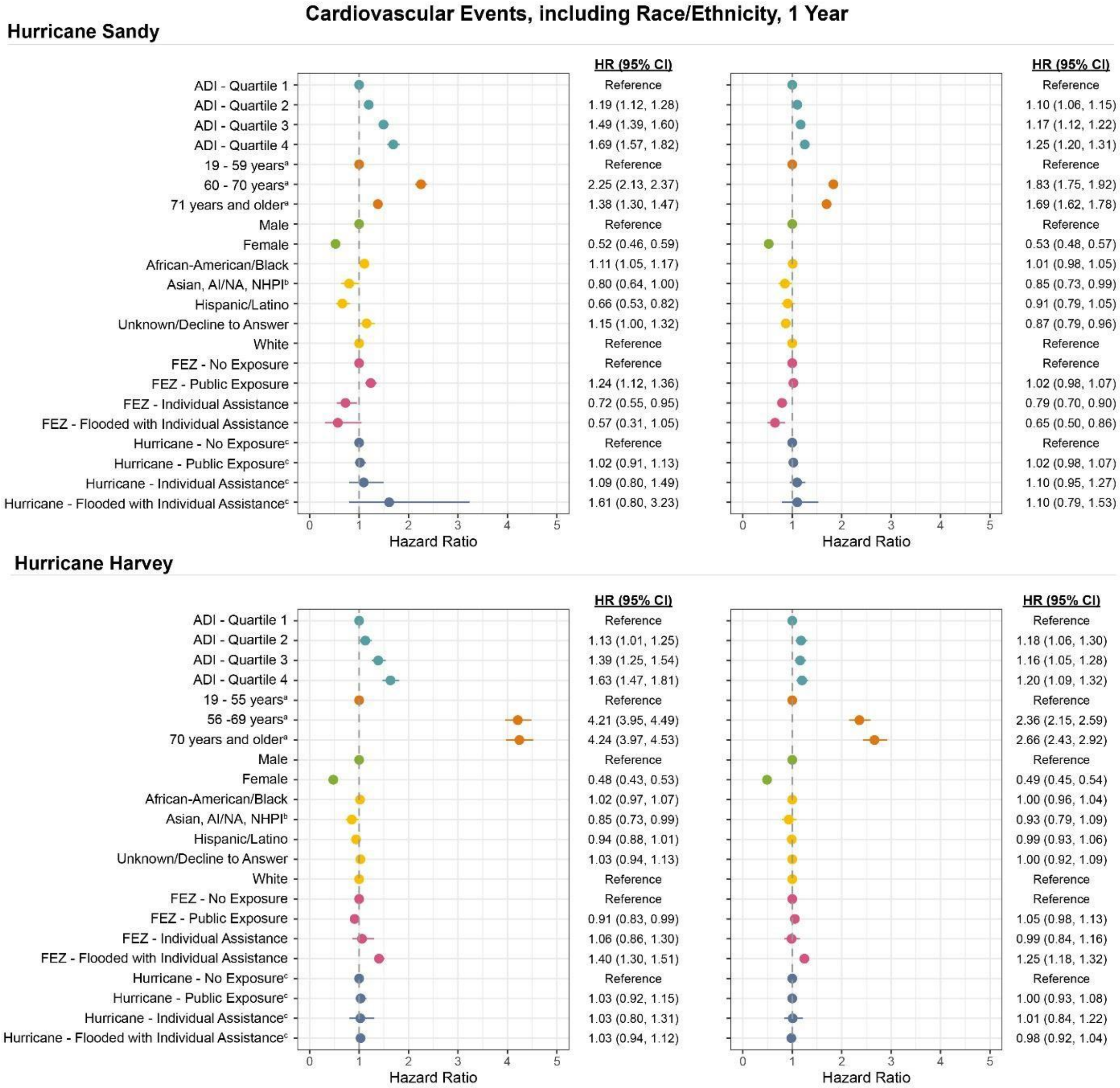


eFigure 6. Hazard ratios (95% confidence intervals) comparing CVEs in various levels of ADI (quartiles), age (tertiles), sex, race/ethnicity (self-reported), FEZ (Future Exposure Zone) and hurricane exposure regions, and stratified by CAN score (< 80, ≥ 80) for 6-months post hurricane compared to the analogous time period from the previous year.

[a] Age ranges for each hurricane differ as each cohort's age was divided into tertiles.

[b] AI/NA = American Indian, Alaska Native; NHPI = Native Hawaiian and Pacific Islander

[c] The lower four values are ratios of post-hurricane HR and pre-hurricane HR.

© 2026 Yip CS et al. *JAMA Network Open*.

eFigure 7. Respiratory Events, Including Race/Ethnicity, 1 Year

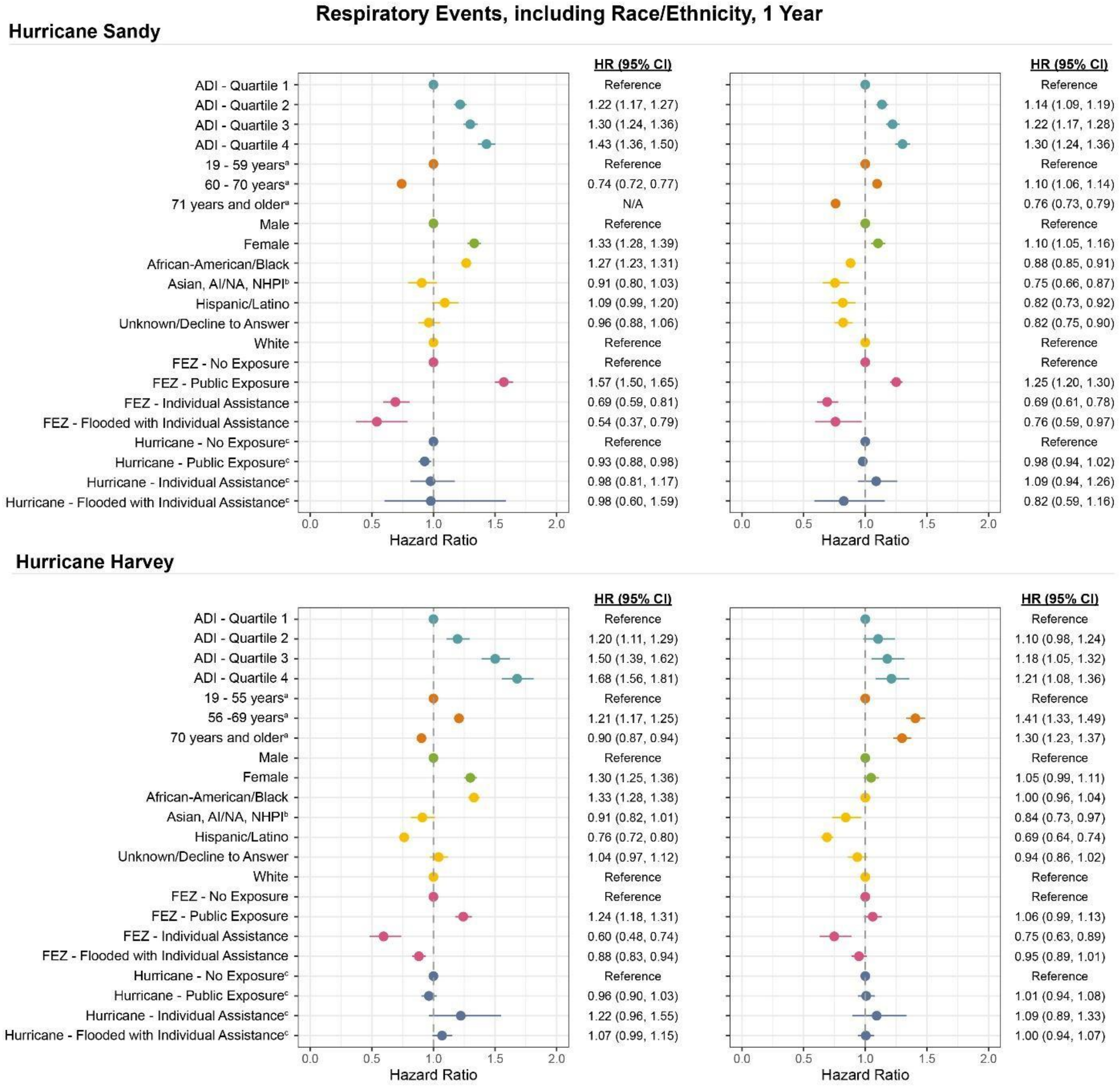


eFigure 7. Hazard ratios (95% confidence intervals) comparing REs in various levels of ADI (quartiles), age (tertiles), sex, race/ethnicity (self-reported), FEZ (Future Exposure Zone) and hurricane exposure regions, and stratified by CAN score (< 80, ≥ 80) for 6-months post hurricane compared to the analogous time period from the previous year. No applicable data (N/A) is shown when there were insufficient events in that strata.

[a] Age ranges for each hurricane differ as each cohort's age was divided into tertiles.

[b] AI/NA = American Indian, Alaska Native; NHPI = Native Hawaiian and Pacific Islander

[c] The lower four values are ratios of post-hurricane HR and pre-hurricane HR.

© 2026 Yip CS et al. *JAMA Network Open*.

eAppendix. Code Stubs and Variable Definitions

Code for main model – SAS Enterprise Guide 8.3

```
PROC PHREG data=sampledata covs(aggregate);
        class adi_quartile (ref = "1") age_tertile (ref = "1") fez (ref = "0") sex (ref="0") hurricane (ref = "0")
        model (tstart, tstop) * status (0 1) = adi_quartile age_tertile sex fez fez*hurricane / ties=breslow;
        id patientid;
run;
```

ADI:

```
case when adi <= '25' then 1
        when adi > '25' and adi <= '50' then 2
        when adi > '50' and adi <= '75' then 3
        when adi > '75' then 4
end as adi_quartile;
```

Age:

```
select age,
ntile(3) over (order by age asc) as age_tertile
from sampledata
order by age asc;
```

Race:

```
Add race1 as (case when race = 'White' and ethnicity = 'not Hispanic or Latino' then 0
                when race = 'White not of Hisp orig' then 0
                when race = 'Black or African American' then 1
                when race = 'White' and ethnicity = 'Hispanic or Latino' then 2
                when ethnicity = 'Hispanic or Latino' then 2
                when race = 'Asian' or race = 'American Indian or Alaska Native' or race = 'Native Hawaiian or Other Pacific Islander' then 3
                when race = 'Unknown by Patient' or race = 'Declined to Answer' then 4
        end);
```

CAN:

```
data sampledata;
set healthydata;
where can LT 80;

data sampledata;
set sickdata;
where can GE 80;
```

© 2026 Yip CS et al. *JAMA Network Open*.

eFigure 8. STROBE Cohort Exclusion Diagram

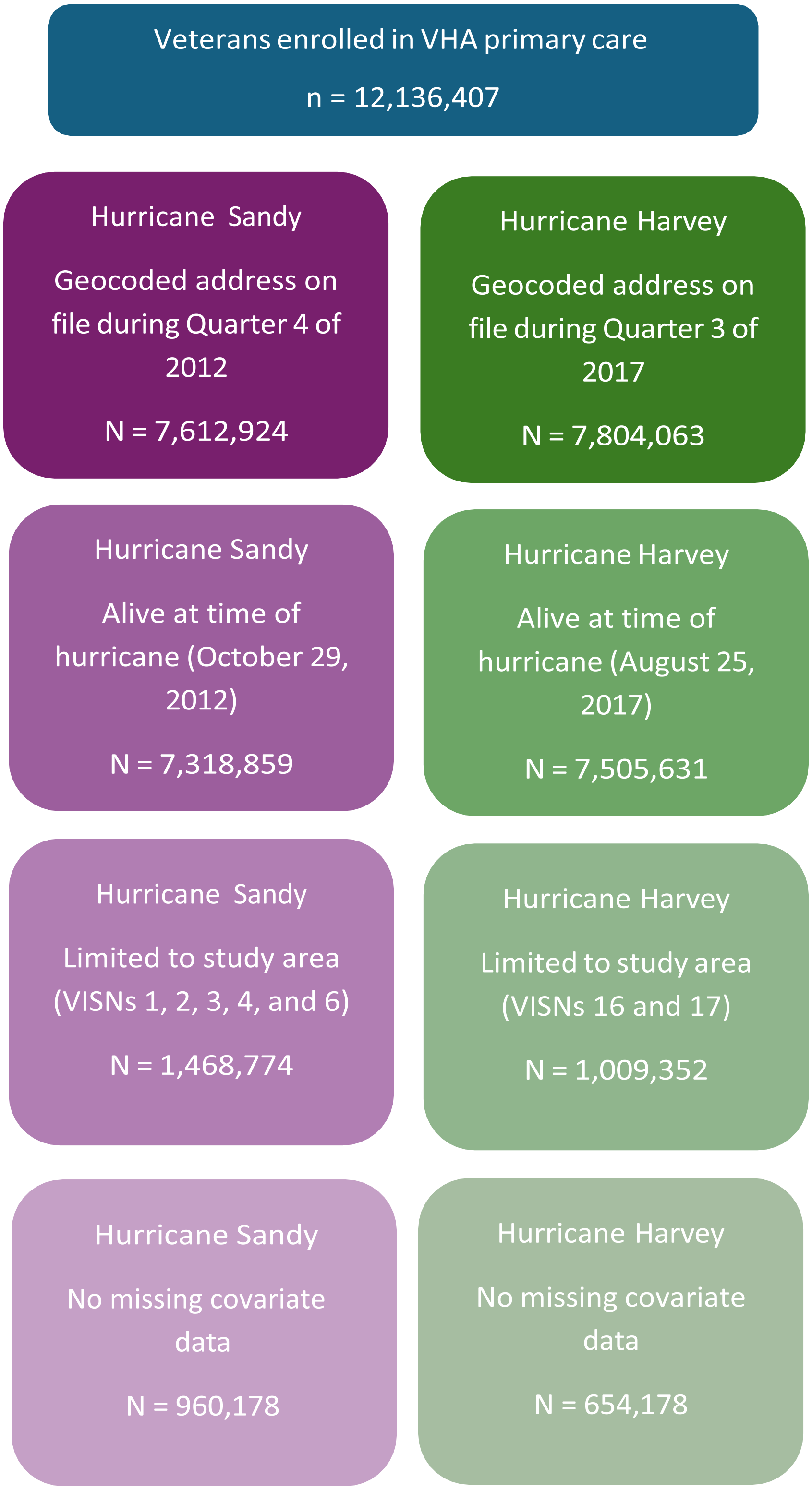


© 2026 Yip CS et al. *JAMA Network Open*.

| | Hurricane Sandy Cohort Before Exclusions N = 1,468,774 | Hurricane Sandy Analytical Cohort N = 960,178 |
|---|---|---|
| ADI Quartile 1 (%) | 18.13 | 17.51 |
| ADI Quartile 2 (%) | 33.18 | 33.21 |
| ADI Quartile 3 (%) | 29.12 | 29.43 |
| ADI Quartile 4 (%) | 19.57 | 19.85 |
| Mean ADI (SD) | 50 (25) | 51 (25) |
| Mean Age (SD) | 62 (17) | 63 (16) |
| Male (%) | 92.94 | 93.30 |
| Female (%) | 7.06 | 6.70 |
| | Hurricane Harvey Cohort Before Exclusions N = 1,009,352 | Hurricane Harvey Analytical Cohort N = 654,178 |
| ADI Quartile 1 (%) | 6.77 | 5.82 |
| ADI Quartile 2 (%) | 25.89 | 24.29 |
| ADI Quartile 3 (%) | 35.21 | 34.40 |
| ADI Quartile 4 (%) | 32.13 | 35.49 |
| Mean ADI (SD) | 61 (23) | 63 (23) |
| Mean Age (SD) | 59 (17) | 60 (16) |
| Male (%) | 90.05 | 90.04 |
| Female (%) | 9.95 | 9.96 |

eTable 2. Full Cohort v. Analytical Cohort Comparison

© 2026 Yip CS et al. *JAMA Network Open*.
