# Supplementary material for: Hurricanes, Neighborhood Disadvantage, and Cardiopulmonary Health in US Veterans
Source: JAMA Netw Open. 2026 Apr 17;9(4):e267830. doi: 10.1001/jamanetworkopen.2026.7830 (PMC13090845; doi:10.1001/jamanetworkopen.2026.7830)
Supplement: Supplement 2. — Data Sharing Statement [file jamanetwopen-e267830-s002.pdf]

Data Sharing Statement

Yip. Hurricanes, Neighborhood Disadvantage, and Cardiopulmonary Health in US Veterans. *JAMA Netw Open*. Published April 17, 2026. doi:10.1001/jamanetworkopen.2026.7830

Data
Data available: No

Additional Information
Explanation for why data not available: The data are available from the Veterans Health Administration with approval of a data use agreement: https://www.research.va.gov/programs/vinci/
